# Burden of mild and moderate atopic dermatitis in adults: results from a real-world study in the United States

**DOI:** 10.1007/s00403-025-03910-y

**Published:** 2025-03-12

**Authors:** Jonathan I. Silverberg, Peter Anderson, Joseph C. Cappelleri, James Piercy, Mark E. Levenberg, Daniela E. Myers, Robert A. Gerber

**Affiliations:** 1 George Washington University School of Medicine and Health Sciences, Washington, District of Columbia, USA; 2 Adelphi Real World, Bollington, UK; 3 Statistical Research and Data Science Center, Pfizer Inc, Groton, CT USA; 4 Medical Affairs, Pfizer Inc, Collegeville, PA USA; 5 HTA, Value and Evidence, Pfizer Inc, 500 Arcola Road, D4344, Collegeville, PA 19426 USA; 6 HTA, Value and Evidence, Pfizer Inc, Groton, CT USA

**Keywords:** Eczema, Atopic dermatitis, Real world data, Burden of disease

## Abstract

Few studies explore the burden of mild-to-moderate atopic dermatitis (AD). We aimed to investigate disease burden in mild-to-moderate AD using real-world data from adults with AD and their physicians in the United States. Data were drawn from the Adelphi Real World AD Disease Specific Programme™, a cross-sectional survey of physicians and their patients with AD in real-world clinical practice in the US from November 2014 to February 2015. Physicians provided data for the next five eligible adults who consulted the physician. Patients had a physician-reported history of moderate-to-severe AD. Overall, 284 and 554 adults with physician-perceived mild or moderate AD at the most recent consultation, respectively, were included in the analysis. Patients with moderate AD experienced more flares (*p* <.001) and had dry skin, pruritus, and cracking/raw skin day-to-day that were more severe (*p* <.0001) and when experiencing a flare (*p* <.05) than patients with mild AD. Adults with either mild or moderate AD used a similar number of treatments. Patients with moderate AD reported greater impact on health status, health-related quality of life, and productivity than those with mild AD. Adults with mild-to-moderate AD experienced substantial daily impact from symptoms despite multiple therapies. Unmet needs remain and more can be done to improve disease control in adults with mild-to-moderate AD.

**Supplementary Information:**

The online version contains supplementary material available at 10.1007/s00403-025-03910-y.

## Introduction

Atopic dermatitis (AD) is a chronic inflammatory disease characterized by eczematous lesions and pruritus [4, 7, 19, 36]. In the United States, the prevalence of AD is 4.9-10.2% among adults [6, 11, 29, 34] and 14.8-24% among children (< 18 years) [1, 23, 28]. AD is associated with numerous comorbidities, including asthma, allergies, anxiety, depression, autoimmune disease, and cardiovascular conditions [9, 31]. Impaired sleep and fatigue are also commonly reported [27, 38], together with impaired health-related quality of life (HRQoL) [16, 27, 32]. Adults with AD may have impairments in HRQOL and productivity similar to those with psoriasis [18]. In a US cross-sectional, population-based study, AD resulted in higher total loss in quality-adjusted life-years (QALYs) in adults than other common health disorders, such as autoimmune disorders, food allergy, and heart disease, with greatest QALY loss in females with moderate AD and males with mild AD [30].

The economic burden of AD is significant, with healthcare resource utilization and costs for US adults with AD significantly greater than for those with psoriasis [17]. AD-related healthcare costs in adults, adjusted to 2015 US dollars, were US$5.3 billion, excluding costs of over-the-counter products, presenteeism, or absenteeism [16]. It was estimated that employed adults with AD in the US experience approximately 3 times the level of absenteeism compared with matched controls and report that more than 25% of their work time is missed or rendered ineffective because of their health [18].

Most individuals with AD have mild-to-moderate disease, with severe AD representing a small proportion of AD cases [6]. US surveys showed that only 7-11% of adults with AD have severe disease [11, 31, 32]. Despite this, the focus of much research is on moderate and/or severe AD with little real-world evidence examining patient perceptions alongside physician-reported clinical outcomes in AD. This study investigated AD burden using real-world US data from adults with AD and their physicians, with a focus on mild-to-moderate AD.

## Materials and methods

### Study design

Analyses were performed using data from the Adelphi Real World AD Disease Specific Programme (DSP)™ conducted in the United States from November 2014 to February 2015. DSP is a cross-sectional survey of physicians and their patients; DSP methodology was published previously [2]. Participating physicians could be specialists (e.g., dermatologists, allergists, immunologists) or primary care physicians and were required to have ≥ 3 years of experience treating adults with AD.

Physicians completed a patient record form (PRF) for the next 5 eligible adults who consulted the physician. To be eligible, patients had to be aged *≥* 18 years; have mild, moderate, or severe AD (as determined by the physician based on their subjective assessment informed by available patient records and first-hand observations of, and discussions with, the patient on the day of consultation); and have a history of moderate-to-severe AD and could not be currently involved in a clinical trial for AD. There were no restrictions regarding treatment. PRF data collected included current prescription AD therapies, symptoms, number of flares (acute episodes) in the past 12 months, body regions and percentage of treatable body surface area (%BSA) affected, clinician’s subjective assessment of global AD severity (mild, moderate, severe) currently and when current treatment was initiated, and all components of the Eczema Area and Severity Index (EASI). Patients were invited to complete a patient self-complete form (PSC), which included several patient-reported outcomes (PROs).

### Patient-reported outcome assessments

PROs were assessed using the generic EuroQoL 5-dimension questionnaire (EQ-5D-3L) [24], HRQoL with Dermatology Life Quality Index (DLQI) [20], disease severity with Patient-Oriented Eczema Measure (POEM) [10], and productivity using Work Productivity and Activity Impairment (WPAI) questionnaire [25]. Additional details are provided in the Supplementary Methods.

### Statistical analysis

Analysis included only adults whose AD condition was mild or moderate at the time of data collection. Descriptive analysis was performed and stratified by AD severity. Mean and standard deviation were calculated for continuous variables, and frequency count and percentage were determined for categorical variables. Bivariate analysis was used to compare patients with mild AD and those with moderate AD.

Propensity score analysis was also conducted using inverse probability–weighted regression adjustment to calculate propensity scores for outcomes adjusting for potential confounders (eTable 1 in Supplementary materials), assuming 2 independent groups [26]. Additional details about propensity score analysis methodology [3, 5, 14] are described in the Supplementary Methods. All analyses were performed using Stata (version 15.1 or later).

## Results

### Demographics and clinical characteristics

Participating US physicians (*N* = 199; dermatologists, *n* = 73 [37%]; internal medicine specialists = 52 [26%]; allergists/immunologists = 25 [13%]; primary care physicians = 49 [25%]) completed PRFs for 936 adults: 284 with a current physician-reported assessment of mild AD and 554 with moderate AD. A total of 521 patients (62.2%) completed PSCs, of whom 161 (31%) and 360 (69%) had mild and moderate AD, respectively.

Demographics were similar between adults with mild AD and those with moderate AD, although a lower proportion of those with moderate AD were employed full-time versus those with mild AD (*p* <.05) (Table 1).

**Table 1:** Demographics and AD duration

	Mild AD *n* = 284	Moderate AD *n* = 554	*p* Value^a^
Male, n (%)	127 (44.7)	253 (45.7)	0.9999
Age, mean (SD), y	41.0 (15.7)	39.5 (16.0)	0.1898
White/Caucasian, n (%)	215 (75.7)	419 (75.6)	0.5562
Employed full time, n (%)	183 (65.4)	300 (56.2)	0.0263
BMI, mean (SD), kg/m^2^	26.1 (4.6)	26.6 (4.6)	0.1653
Time since diagnosis, y			
*n*	231	427	–
Mean (SD)	10.0 (10.9)	11.2 (12.4)	0.2250

Abbreviations: AD, atopic dermatitis, BMI body mass index

^a^Patients with mild AD compared with patients with moderate AD

**Fig. 1:**
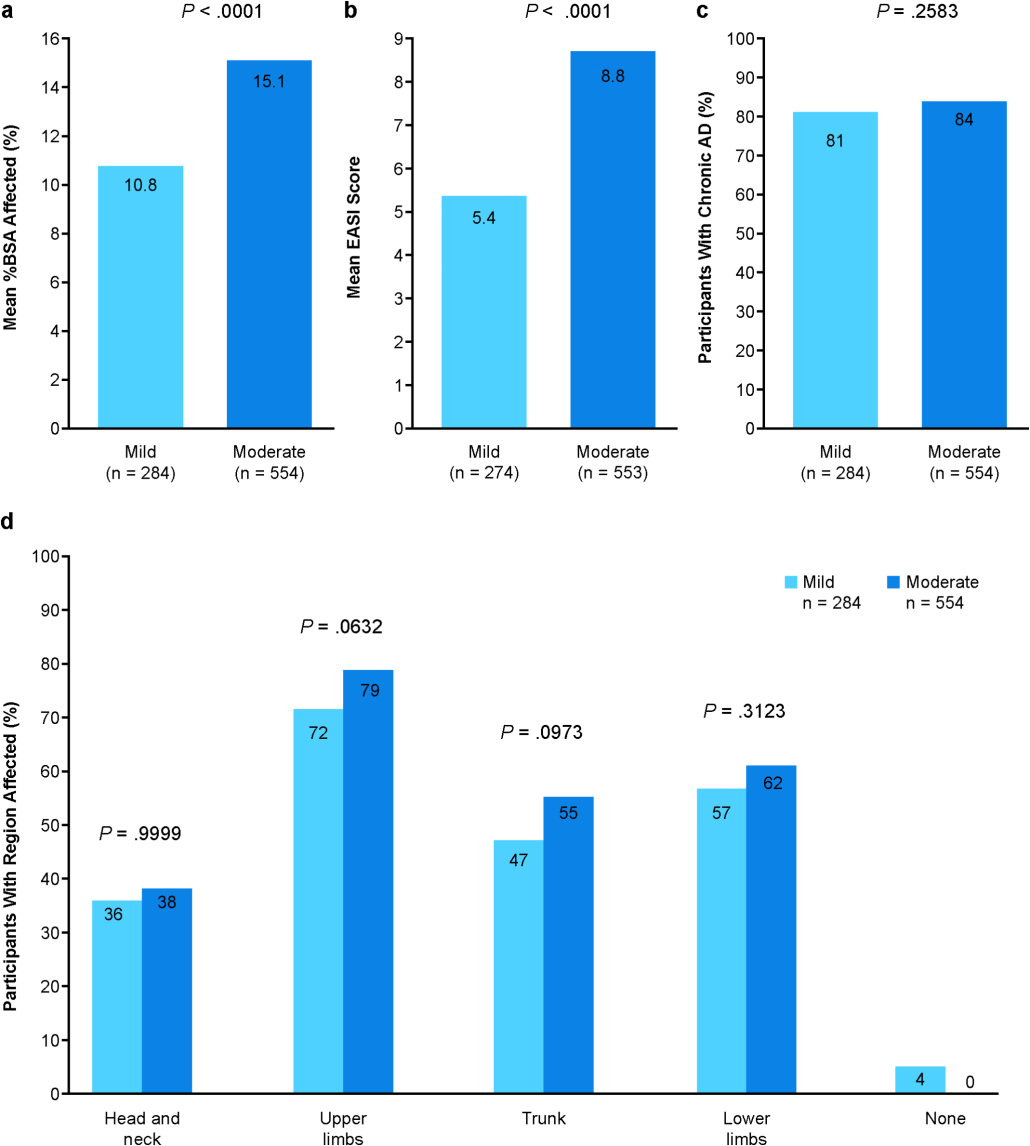
Clinical Characteristics. %BSA affected (**a**); EASI score (**b**); chronic AD (**c**)^a^; body regions affected (**d**). ^a^AD considered chronic by physician. *Abbreviations: %BSA*,* percentage of treatable body surface area; AD*,* atopic dermatitis; EASI*,* Eczema Area and Severity Index*

Mean %BSA affected and mean EASI scores were significantly higher in adults with moderate AD versus mild AD (*p* <.001). Similar proportions of those with mild and moderate AD were considered to have chronic AD by their physicians (Fig. 1a-c). For all body regions, greater proportions of patients with moderate AD were reported to be affected than those with mild AD, but none of these differences were statistically significant (Fig. 1d).

After propensity matching, adults with moderate AD had significantly greater %BSA affected than those with mild AD (*n* = 365, 15.7% vs. *n* = 231, 10.2%, respectively; *p* <.0001) and higher EASI scores (*n* = 427, 8.6 vs. *n* = 221, 5.4, respectively; *p* <.0001). A greater proportion of patients had affected upper (*n* = 365, 79% vs. *n* = 231, 71%, respectively; *p* <.05) and lower limbs (*n* = 365, 63% vs. *n* = 231, 55%, respectively; *p* =.05); however, there was no difference in the proportion of patients with chronic AD (*n* = 427, 82% vs. *n* = 231, 79%, respectively; *p* =.297).

### Comorbidities, atopic conditions, flares, and symptoms

**Fig. 2:**
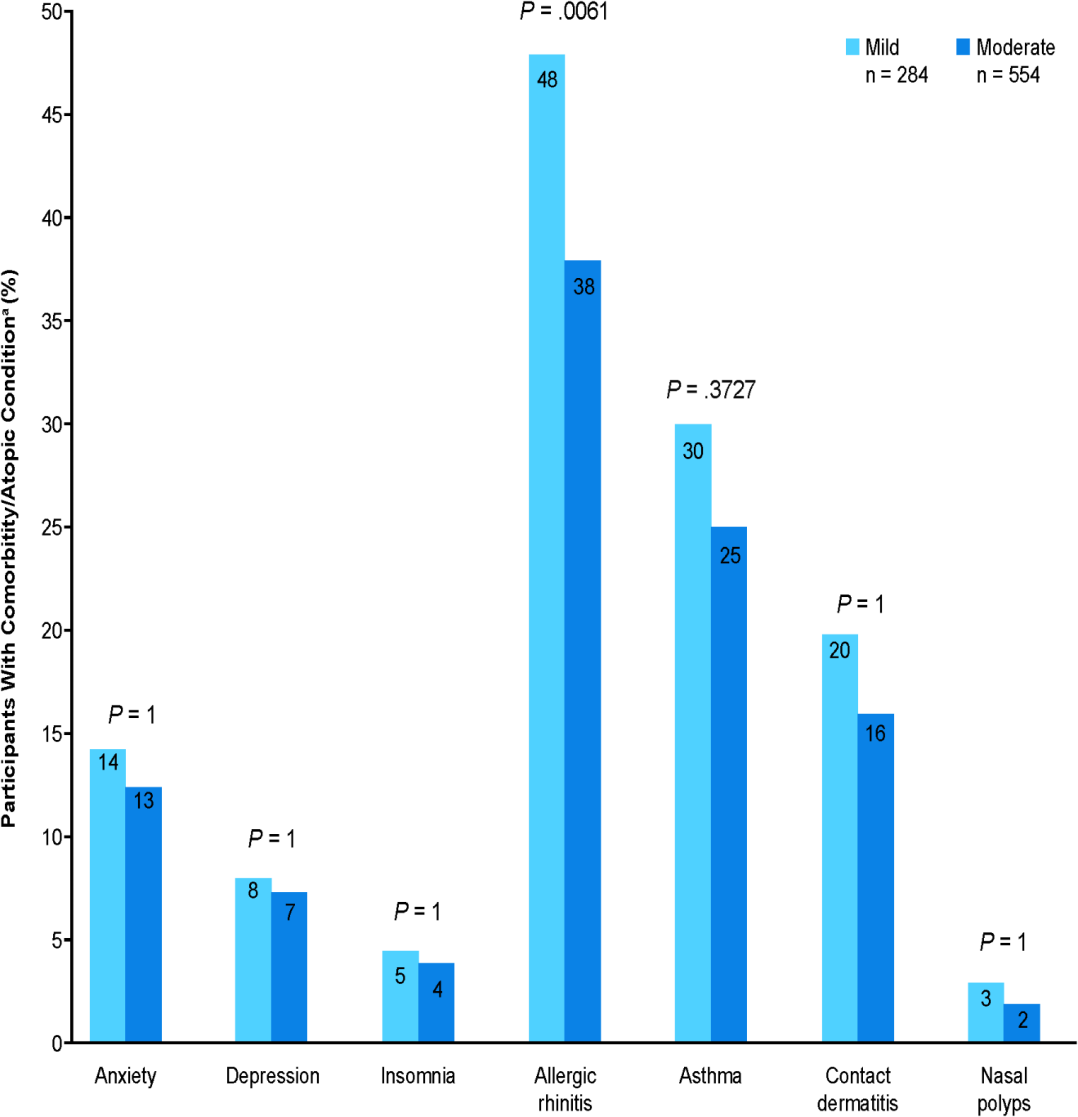
Current Comorbidities and Atopic Conditions. Patients could have multiple comorbidities and atopic conditions; therefore, values do not add up to 100%

**Fig. 3:**
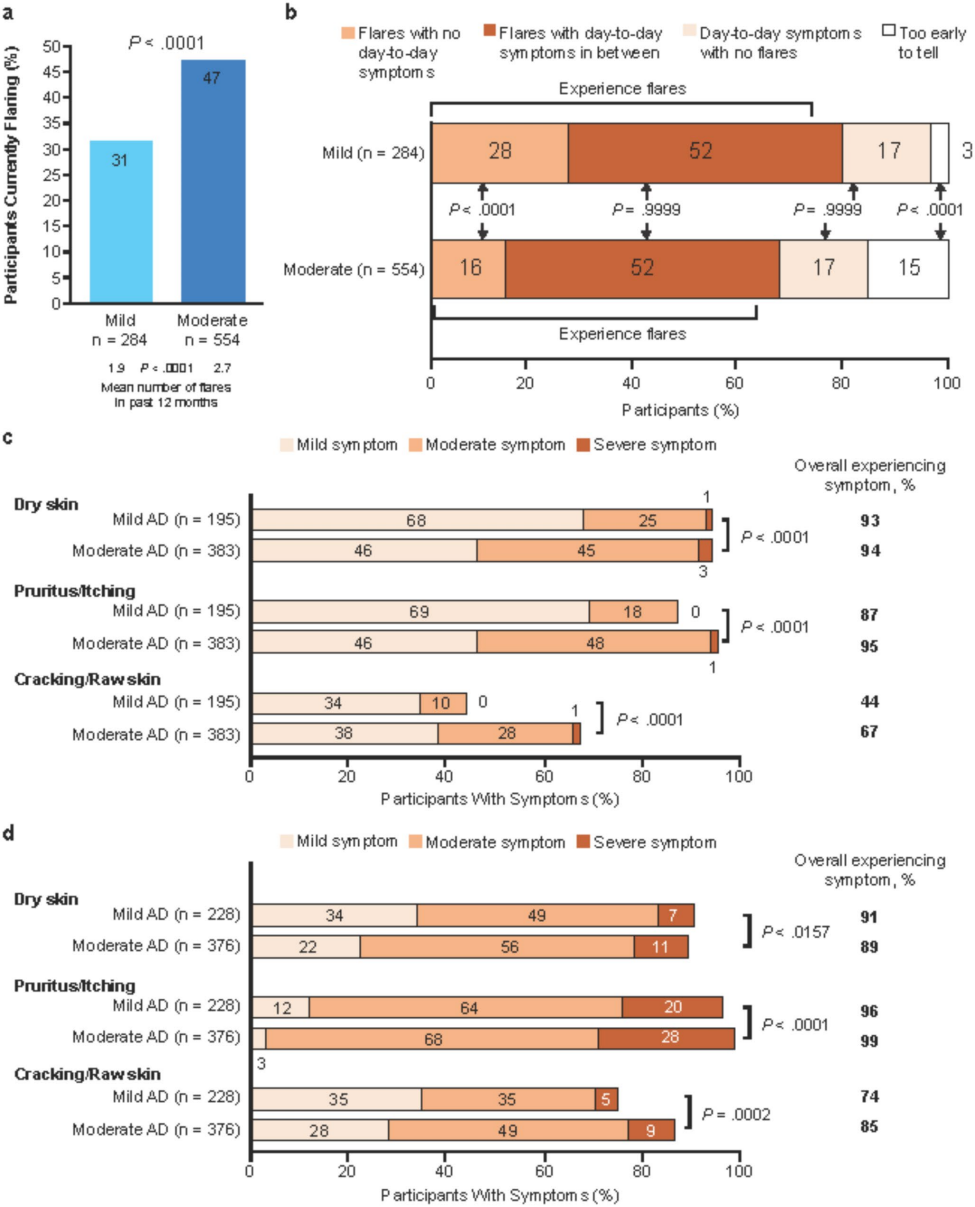
Flares and Day-to-Day Symptoms. Flares (**a**), flares and day-to-day symptoms (**b**), day-to-day symptoms outside of a flare (**c**), symptoms during flare (**d**). *Abbreviations: AD*,* atopic dermatitis*

A higher proportion of adults with mild than with moderate AD experienced allergic rhinitis (*p* <.01) (Fig. 2). A greater proportion of adults with moderate than mild AD experienced a physician-reported flare at the time of data collection (*p* <.0001), and patients with moderate AD experienced more flares in the previous 12 months than those with mild AD (mean *p* <.0001) (Fig. 3a). However, adults with mild versus moderate AD were more likely to have a disease pattern that had included flares at some point in their disease history (80% vs. 68%, respectively) and, particularly, flares with no day-to-day symptoms (*p* <.0001) (Fig. 3b).

After propensity matching, a greater proportion of patients with moderate AD (46%; *n* = 365) experienced a flare at the time of data collection than those with mild AD (31%; *n* = 231) (p *<.*0001). Propensity analysis showed individuals with moderate AD had more symptoms day-to-day (i.e., when not flaring) than those with mild AD (moderate: *n* = 365, mean number of symptoms, 5.5; mild: *n* = 231, mean number of symptoms, 3.2; *p* <.0001). The number of symptoms during a flare was comparable for the 2 groups (moderate: *n* = 355, mean number of symptoms, 6.8; mild: *n* = 231, mean number of symptoms, 6.3; p *=.*2100).

Outside of a flare (on a day-to-day basis), patients with moderate AD and mild AD experienced a mean of 4.7 and 3.5 AD symptoms (of 17 symptoms provided in a list), respectively, of which dry skin, pruritus, and cracking/raw skin were the most common (Fig. 3c). Patients with moderate AD experienced greater severity of these 3 symptoms than those with mild AD (p *<.*0001) (Fig. 3c). When patients were grouped by %BSA affected (0%, > 0% to < 16%, ≥ 16% to < 40%, ≥ 40%) [13] rather than physician assessment of severity, the occurrence and, generally, the severity of dry skin, pruritus, and cracking/raw skin day-to-day increased with increasing %BSA affected (p *<.*01) (eFig. S1).

During a physician-reported flare, patients with mild-to-moderate AD experienced a mean number of 6.3 symptoms (of 17 possible symptoms); the most common symptoms were dry skin, pruritus, and cracking/raw skin (Fig. 3d). Patients with moderate AD were more likely to experience more-severe dry skin (p *<.*05), pruritus, (p *<.*0001), and cracking/raw skin (*p* <.001) (Fig. 3d).

### Control of condition

Physicians considered overall AD to be improving or stable since initiation of the patient’s current treatment regimen for 94% and 62% of patients with mild and moderate AD, respectively; changeable or deteriorating slowly for 6% and 25%; and deteriorating rapidly or totally uncontrolled for 7% and 29% (eFig. S2).

The proportion of patients at each level of control was significantly different between those with mild and moderate AD (*p* <.01). Physicians reported greater satisfaction with the level of control in adults with mild AD versus moderate AD (*p* <.0001); physicians were very or extremely satisfied with the level of control for 46% and 21% of patients with mild and moderate AD, respectively.

### Treatments

Patients with mild and moderate AD were receiving similar numbers of AD therapies (eFig. S3a); however, propensity score analysis showed those with moderate AD used significantly more treatments than those with mild AD (moderate: *n* = 365, mean number of treatments, 2.20; mild: *n* = 231, mean number of treatments, 1.95; *p* <.05).

Patients with mild and moderate AD were on their current regimen for about the same length of time (eFig. S3b). Individuals with moderate AD were more likely to receive oral corticosteroids, oral antibiotics, and analgesics than those with mild AD, whereas patients with mild AD were more likely to be taking a low-potency topical corticosteroid (eFig. S3c). Propensity score analysis showed a greater proportion of patients with moderate versus mild AD received oral corticosteroids (moderate: *n* = 365, 23%; mild: *n* = 231, 9%; *p* <.0001) and phototherapy (moderate: *n* = 365, 13%; mild: *n* = 231, 7%; *p* <.05).

### Severity and assessment of control

Overall, 33% of patients had less severe AD, according to their physicians, and 2% had more severe AD at the time of data collection than at initiation of their current therapy regimen (eFig. S4). For patients with moderate AD at initiation of their current treatment, no significant differences were seen between those considered by their physician to have mild AD at the time of data collection (improved patients) and those with moderate AD (non-improved patients) in the types of treatments received (when considering only treatments taken for > 3 months), with the exception of analgesics (taken by 10% of non-improvers but only 2% of improvers, *p* <.01). Improvers had numerically greater %BSA affected before treatment initiation than non-improvers (26.0% vs. 23.6%) and had a significantly greater reduction in %BSA affected from the start of treatment to the time of data collection (− 16.0% vs. − 9.8%, *p* <.001). Physicians reported greater satisfaction with disease control for patients with mild AD at the time of data collection (improvers) than for those with moderate AD (non-improvers) (*p* <.0001). In response to whether the current level of disease control was the best that could be achieved, physicians thought better control could be achieved in 56% of patients with moderate AD and in 33% of those with mild AD (*p* <.0001).

### Impact on patient

**Fig. 4:**
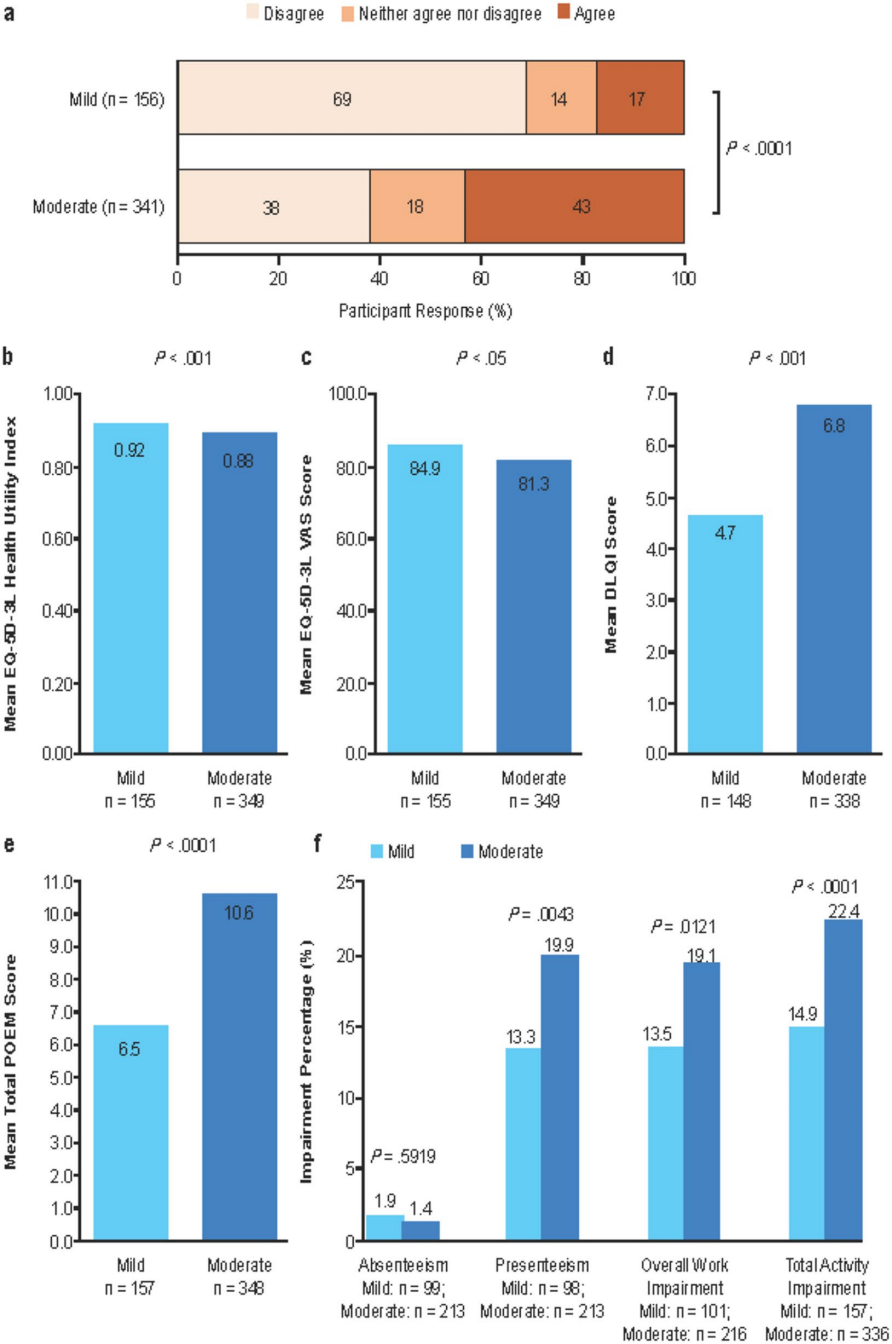
Patient-Reported Impact. Emotional upset^a^ (**a**), EQ-5D-3L utility index (**b**), EQ-5D-3L VAS score (**c**), DLQI score (**d**), POEM score (**e**), WPAI score (**f**). ^a^Response to statement “My AD condition is a major problem in everyday life and causes considerable emotional upset” *Abbreviations: DLQI*,* Dermatology Life Quality Index; EQ-5D-3L*,* EuroQoL 5-dimension 3-level; POEM*,* Patient-Oriented Eczema Measure; VAS*,* visual analog scale; WPAI*,* Work Productivity and Activity Impairment*

Demographic characteristics were comparable between patients who completed a PSC and those who did not. More patients completing a PSC with moderate versus mild AD felt AD was a major problem in everyday life causing them considerable emotional upset (*p* <.0001) (Fig. 4a).

All PROs showed statistical differences between individuals with mild and moderate AD in bivariable analyses (Fig. 4b-e) and propensity score analyses; for EQ-5D-3L, moderate AD, *n* = 231 and health utility index 0.88; mild AD, *n* = 119 and health utility index = 0.91; *p* <.05; for DLQI, moderate AD n *=* 224 and total score = 6.84; mild AD, *n* = 111 and total score = 4.60; *p* <.0001; for Patient-Oriented Eczema Measure, moderate AD, *n* = 232 and total score = 10.48; mild AD, *n* = 119 and total score = 6.25; *p* <.0001.

Adults with moderate versus mild AD reported higher rates of presenteeism (*p* <.01), overall work impairment (*p* <.05), and total activity impairment (*p* <.0001) (Fig. 4f). Total activity impairment, but no other measure of productivity, was also statistically greater in those with moderate AD than in those with mild AD by propensity analysis (moderate: *n* = 219, impairment = 22.2%; mild: *n* = 119, impairment = 15.3%; *p* <.0001).

Based on a rating of 1–7 for satisfaction with disease control (1 being extremely dissatisfied and 7 being extremely satisfied), propensity analysis indicated a significantly lower level of satisfaction with disease control reported by individuals with moderate AD (4.7; *n* = 365) compared with those with mild AD (5.3; *n* = 231) (p *<.*0001). No difference was observed in the number of physician consultations for AD in the previous 12 months between patients with mild and moderate AD (median number of visits = 2.0 for both groups).

More patients with moderate AD than patients with mild AD reported insomnia/sleep disruption due to AD during a flare (40% of 242 vs. 26% of 120, p *<.*05) but not day-to-day (19% of 217 vs. 12% of 89, p *=.*37). More patients with moderate AD than patients with mild AD reported pain/soreness due to AD day-to-day (47% of 217 vs. 17% of 89, p *<.*0001) but not during a flare (58% of 242 vs. 47% of 120, p *=.*087).

## Discussion

In this real-world study of US adults with mild or moderate AD, a substantial disease burden with poorly controlled symptoms, HRQOL impairment, and impact on daily life was observed despite many patients receiving standard-of-care treatment. Moderate AD imposed a greater symptom burden than mild AD, largely because of the severity of symptoms, with the frequency of symptoms consistently high in both mild and moderate AD. Approximately 1 in 3 individuals with mild AD and 1 in 2 individuals with moderate AD experienced a flare at the time of data collection. Adults with mild and moderate AD experienced an average of 1.9 and 2.7 flares, respectively, in the previous 12 months, which is considerably lower than the 9.6 flares per year reported by adults with moderate-to-severe AD in a large international study [39]. However, this comparison must be interpreted with caution because the flare incidences in the past year were evaluated by physicians in the current study and by patients in the previous study. Physician perception of disease control and satisfaction with level of control were greater for patients with mild AD versus those with moderate AD, and more than two-thirds of those who were perceived by their physician to have moderate AD when they started their current treatment were not considered to have experienced improvement in their AD.

At approximately the same time as our study, *Boguniewicz et al.* [8] provided treatment recommendations for moderate-to-severe AD patients addressing current and emerging therapies identifying the presence of treatment failure. The authors were suggesting that definition of treatment failure would take into consideration one or more of the following: Inadequate clinical improvement, failure to achieve stable long-term disease control, presence of ongoing impairment (including pruritus, pain, loss of sleep, and poor quality of life) while on treatment, unacceptable adverse events or poor tolerability experienced with the treatment. At that time, the authors recommended wider use of dupilumab in patients experiencing treatment failure. A more recent consensus document [21] provided further advice for the management of these patients, in particular regarding the place in therapy of JAK inhibitors in difficult-to-treat subpopulations such as those with head, neck and hand involvement [12, 37] and those with multiple comorbidities and concomitant medications. The recommendations from these consensus statements may be appropriate to address the unmet needs identified in the moderate patients in our study since these patients often continue to experience a significant symptom and disease burden.

A substantial proportion of patients reported AD to be a major problem causing them considerable emotional upset, with 1 in 6 patients with mild AD and slightly more than 1 in 2 patients with moderate AD reporting this to be the case. Patients with moderate AD reported greater impact of disease than those with mild AD on all PRO endpoints. Mean EQ-5D-3L utility index scores were 0.88 and 0.92 and mean EQ-5D-3L visual analog scale (VAS) scores were 81.3 and 84.9 for patients with moderate AD and those with mild AD, respectively, versus 0.83 for the EQ-5D-3L utility index and 80.0 for the EQ-5D-3L VAS in the general US population [35]. These findings suggest little impact of mild-to-moderate AD on overall health status, as measured by the EQ-5D-3L. It is likely much of the impact on HRQOL from AD is not adequately reflected in the EQ-5D index/VAS, which has a recall of “today” and, subsequently, may not capture the waxing and waning course and variable impact of AD on patient lives day-to-day. By contrast, the mean DLQI score of 4.7 and 6.8 for patients with mild AD and those with moderate AD, respectively, indicates mild or moderate disease had a substantial impact on HRQOL compared with the score of 1.1 reported for controls without AD [32].

Productivity losses in the current study were somewhat lower than those reported from an analysis of the 2017 US National Health and Wellness Survey (NHWS) [33]. For individuals with mild AD in the current study and the NHWS analysis, absenteeism was 2% and 5%, presenteeism was 13% and 20%, and overall work impairment was 14% and 22%, respectively. For individuals with moderate AD in the current study and the NHWS analysis, absenteeism was 1% and 7%, presenteeism was 20% and 27%, and overall work impairment was 19% and 29%, respectively. The different productivity impact reported in these 2 studies might occur, at least in part, because of the subjective classification of disease severity, which was physician defined in the current study but patient-defined in the NHWS analysis, limiting the interpretation of a direct comparison of findings.

The current study offers in-depth insight into a population of adults with mild or moderate AD in the United States who met the eligibility criteria but were not preselected. It provides real-world physician-reported clinical data together with PROs. However, there are limitations. Patients included in the DSP sample were the next 5 eligible adults who consulted the physician; this is a quasi-random sample and might favor those who seek care frequently. Individuals who experienced only mild AD were excluded to ensure all patients had AD and not another skin condition because AD is heterogenous and there are various means of severity classification. Classification of patients as having mild or moderate AD was based on the physicians’ subjective assessment which will take into account medical history together with consideration of the physical and psychological state of the patient observed on the day of the consultation. However, these subjective assessments do correlate well with the clinical EASI score. In our study, mild patients had a mean EASI score of 5.4 and moderate patents a mean of 8.8 (Fig. 1). These figures are consistent with clinical definitions of mild and moderate patients [13, 22]. As with all observational research of this type, data quality depends on the accurate reporting of information by physicians (using available patient records) and patients, for whom recall bias may be impacted. Although minimal exclusion criteria governed the selection of physicians, physician inclusion depends on willingness to participate in research such as this.

## Conclusions

Adults with mild-to-moderate AD experienced substantial daily impact from symptoms despite multiple therapies. Although patients with moderate AD experienced more flares and had dry skin, pruritus, and cracking/raw skin day-to-day that were more severe than patients with mild AD, adults with mild AD underwent a similar number of treatments as those with moderate AD. Patients with moderate AD reported greater impact on health status, HRQOL, and productivity than those with mild AD. Unmet needs remain and novel treatments or better management strategies using available therapies are necessary to provide improved disease control in adults experiencing mild or moderate AD.

## Electronic supplementary material

Below is the link to the electronic supplementary material.

Supplementary Material 1

## Data Availability

All data, i.e. methodology, materials, data and data analysis, that support the findings of this survey are the intellectual property of Adelphi Real World. All requests for access should be addressed directly to Peter Anderson at Peter.Anderson@adelphigroup.com. Peter Anderson is an employee of Adelphi Real World.
