# Supplementary material for: Burden of mild and moderate atopic dermatitis in adults: results from a real-world study in the United States
Source: Arch Dermatol Res. 2025 Mar 12;317(1):556. doi: 10.1007/s00403-025-03910-y (PMC11903610; doi:10.1007/s00403-025-03910-y)

SUPPLEMENTARY MATERIALS

TITLE

Burden of Mild and Moderate Atopic Dermatitis in Adults: Results from a Real-World Study in the United States

Jonathan I. Silverberg, MD, PhD, MPH,[1] Peter Anderson, BSc (Hons),[2] Joseph C. Cappelleri, PhD,[3] James Piercy, MSc,[2] Mark E. Levenberg, DO,[4] Daniela E. Myers, MPH,[5] Robert A. Gerber, PharmD, MA, MBA[6*]

[1]George Washington University School of Medicine and Health Sciences, Washington, District of Columbia, United States; [2]Adelphi Real World, Bollington, United Kingdom; [3]Statistical Research and Data Science Center, Pfizer Inc., Groton, Connecticut, United States; [4]Medical Affairs, Pfizer Inc., Collegeville, Pennsylvania, United States; [5]HTA, Value and Evidence, Pfizer Inc., Collegeville, Pennsylvania, United States; [6]HTA, Value and Evidence, Pfizer Inc., Groton, Connecticut, United States

* Affiliation when research was conducted.

JOURNAL NAME

Archives of Dermatological Research

CORRESPONDING AUTHOR

Daniela E. Myers; Pfizer Inc, 500 Arcola Road, D4344, Collegeville, PA 19426, USA. Tel: +1 646-812-2836

Email: daniela.myers@pfizer.com.

SUPPLEMENTARY METHODS

SUPPLEMENTARY TABLES

eTable1. Propensity Analysis

SUPPLEMENTARY FIGURES

eFig. S1 Day-to-Day Symptoms by %BSA Affected

eFig. S2 Physician Perception of Control

eFig. S3 Treatment. Number of current treatments (a). Duration of current treatments (b). Current treatments (c). Abbreviations: OCS, oral corticosteroid; TCI, topical calcineurin inhibitor; TCS, topical corticosteroid.

eFig. S4 Current Severity Versus Severity at Initiation of Current Treatment

Supplementary Methods

PRO Assessments

The EuroQol 5-dimension, 3-level questionnaire (EQ-5D-3L) is a generic instrument used to assess patient health status that comprises five individual items and a 20-cm vertical visual analog scale (VAS).[6] The individual items ask the respondent to indicate the level of problems related to mobility, self-care, and usual activities (eg, work, study, housework, family, or leisure activities) and the severity of pain/discomfort and anxiety/depression experienced (if any). Each item provides a score ranging from 1 to 3 (no problems, some problems, and extreme problems); a single health utility index score is generated using a country-specific algorithm that provides a number, with 1 indicating perfect health, 0 indicating death, and <0 worse than death.[9] Patients indicate their general health status on the day that they complete the EQ-5D-3L by drawing a line on the VAS to provide a score ranging from 0 (worst imaginable health state) to 100 (best imaginable health state).

The Dermatology Life Quality Index (DLQI) is an instrument designed for use in clinical practice to obtain the patient's perspective of the impact of dermatologic diseases over the past week. It is applicable to atopic dermatitis (AD).[5] The DLQI consists of 10 items, each rated on a 4-point scale (0-3), providing a total composite score in the range of 0-30. Higher DLQI scores indicate poorer health-related quality of life (HRQoL), with a total score of 0-5 indicating mild impairment, 6-10 indicating moderate impairment, and 11-30 indicating severe impairment.[8]

The Patient-Oriented Eczema Measure (POEM) includes 7 questions regarding the frequency of a number of signs and symptoms of eczema in the past week, with each question scored from 0 to 4 (0, no days; 1, 1-2 days; 2, 3-4 days; 3, 5-6 days; 4, every day), resulting in a maximum total score of 28.[3] A total score of 0-7 indicates mild disease, 8-19 indicates moderate disease, and 20-28 indicates severe disease.[8]

The Work Productivity and Activity Impairment (WPAI) questionnaire is used to assess the impact of disease on work productivity and daily activities over the past 7 days. It was completed by patients in a large number of studies and a wide range of disease areas. Because it is not disease specific, it can be used to compare the impact on productivity across diseases. The WPAI is composed of 6 items and results in the generation of 4 scores, each expressed as a percentage of work time missed or a percentage of impairment:[7]

- Absenteeism (work time missed because of impairment), calculated as hours missed as a percentage of total work hours using patient-reported hours missed during the 7-day recall period ([patient-reported hours worked during the 7-day recall period + patient-reported hours missed during the 7-day recall

period] × 100)

- Presenteeism (ability to function at work while being impaired) was calculated as a percentage using patient-reported impact of AD on productivity at work during the 7-day recall period recorded on a scale of 0 (no impact) to 10 (prevented patient from working) × 10
- Overall work impairment: calculated as a percentage using patient-reported hours worked, patient-reported hours missed, and patient-reported impact of AD on productivity during the 7-day recall period, applying an algorithm described on the WPAI website (http://www.reillyassociates.net)
- Total activity impairment: calculated as a percentage using patient-reported impact of AD on productivity in regular unpaid activities during the 7-day recall period recorded on a scale of 0 (no impact) to 10 (prevented activities) × 10

Statistical Analysis

Specific analyses used depended on the outcomes being compared: *t* test (with Welch's correction for unequal variances, if needed) for continuous outcomes and 2 comparator groups, Mann-Whitney *U* test for ordinal outcomes and 2 comparator groups, Pearson χ^2 test for categorical outcomes where the contingency table was larger than 2 × 2, and Fisher exact test for binary outcomes where the contingency table was 2 × 2.[1]

For propensity score analyses, inverse probability–weighted regression adjustment was chosen because it is doubly robust, yielding accurate treatment effect estimates when either the propensity score model or the outcome model is correctly specified.[2] Only patients for whom all relevant data were available and whose data did not violate the overlap assumption[9] were included. The overlap assumption states that each observation has a positive probability of receiving each treatment; any observation that violates this assumption cannot be assigned to one particular group. To compare outcomes stratified by mild and moderate AD, balance was assessed by calculating standardized mean differences; a standardized mean difference between −10% and 10% (not inclusive) was indicative of adequate balance.[4] In addition, convergence had to be achieved.

References

1. Austin PC. (2011). An introduction to propensity score methods for reducing the effects of confounding in observational studies. Multivariate Behav Res.46(3):399-424.

2. Bang H, Robins JM. (2005). Doubly robust estimation in missing data and causal inference models. Biometrics.61(4):962-73.

3. Charman CR, Venn AJ, Williams HC. (2004). The patient-oriented eczema measure: development and initial validation of a new tool for measuring atopic eczema severity from the patients' perspective. Arch Dermatol.140(12):1513-9.

4. Crump RK, Hotz VJ, Imbens GW, Mitnik OA. (2009). Dealing with limited overlap in estimation of average treatment effects. Biometrika.96(1):187-99.

5. Finlay AY, Khan GK. (1994). Dermatology Life Quality Index (DLQI)-a simple practical measure for routine clinical use. Clin Exp Dermatol.19(3):210-6.

6. Rabin R, de Charro F. (2001). EQ-5D: a measure of health status from the EuroQol Group. Ann Med.33(5):337-43.

7. Reilly MC, Tanner A, Meltzer EO. (1996). Work, classroom and activity impairment instruments. Clinical Drug Investigation.11(5):278-88.

8. Silverberg JI, Gelfand JM, Margolis DJ, et al. (2018). Severity strata for POEM, PO-SCORAD, and DLQI in US adults with atopic dermatitis. Ann Allergy Asthma Immunol.121(4):464-8.e3.

9. Szende A, Oppe M, Devlin N. (2007) EQ-5D value sets: Inventory, comparative review and user guide. Dordrecht, Netherlands.ISBN-10 1-4020-5510-2 (HB).

Supplementary Tables

eTable1. Propensity Analysis

**Outcome Variable**	**Confounders**
**Physician-reported**	
Current region affected	Age
Head/neck	Sex
Upper limbs	Body mass index
Trunk	Time since diagnosis
Lower limbs	Current flaring status (when not deriving current flaring status as an outcome)
Current %BSA affected	Charlson Comorbidity Index
Current flaring status	
Number of symptoms during flare	
Number of baseline symptoms	
Number of current treatments	
Current treatment	
Topicals	
Systemic steroids	
Systemic immunosuppressants	
Phototherapy	
Wet wraps	
Bleach baths	
Antihistamines	
Antibiotics	
**Patient-reported**	
Current satisfaction with control	Age
EQ-5D-3L utility index	Sex
EQ-5D-3L VAS score	Body mass index

DLQI	Time since diagnosis
POEM total score	Current flaring status
WPAI	Charlson Comorbidity Index
Absenteeism	Current treatment
Presenteeism	Topicals
Overall work impairment	Systemic steroids
Total activity impairment	Systemic immunosuppressants
	Phototherapy
	Wet wraps
	Bleach baths

Abbreviations: %BSA, percentage of treatable body surface area; DLQI, Dermatology Life Quality Index; EQ-5D-3L, EuroQoL 5-dimension, 3-level questionnaire; POEM, Patient-Oriented Eczema Measure; WPAI, Work Productivity and Activity Impairment; VAS, visual analog scale.

Supplementary Figures

eFig. S1 Day-to-Day Symptoms by %BSA Affected

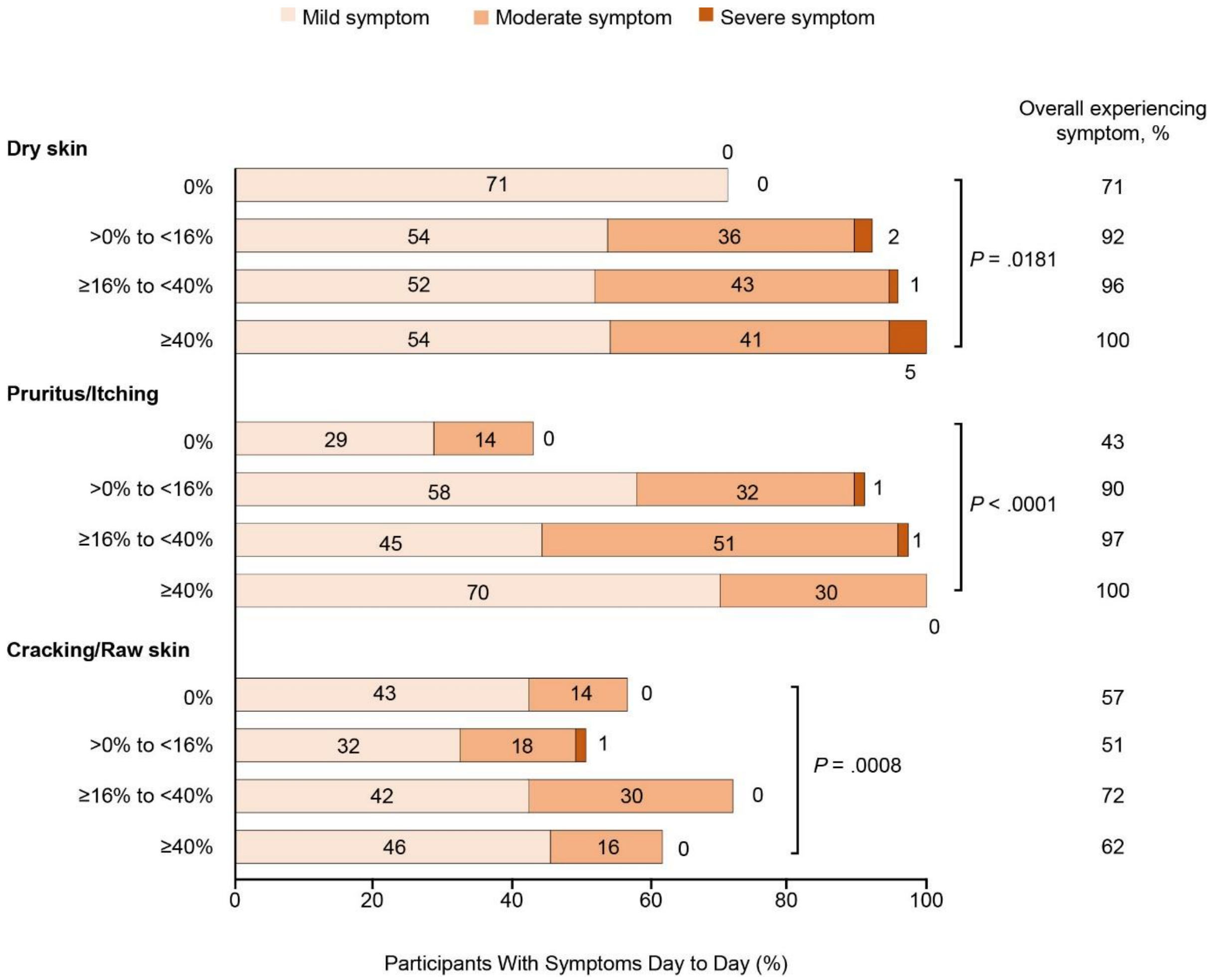

Abbreviations: %BSA, percentage of treatable body surface area.

eFig. S2 Physician Perception of Control

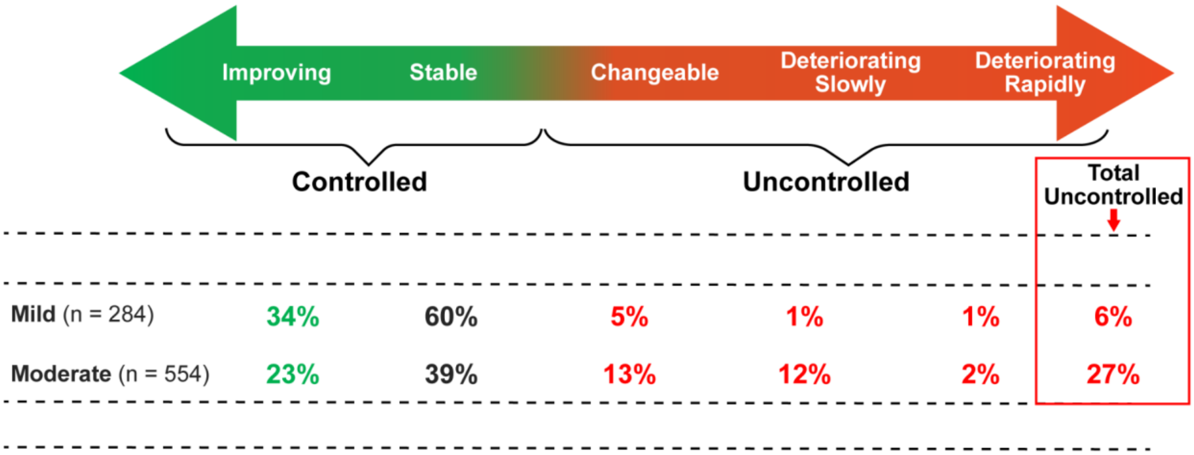

	Improving	Stable	Changeable	Deteriorating Slowly	Deteriorating Rapidly	Total Uncontrolled
**Mild** (n = 284)	34%	60%	5%	1%	1%	6%
**Moderate** (n = 554)	23%	39%	13%	12%	2%	27%

eFig. S3 Treatment. Number of current treatments (a). Duration of current treatments (b). Current treatments (c).

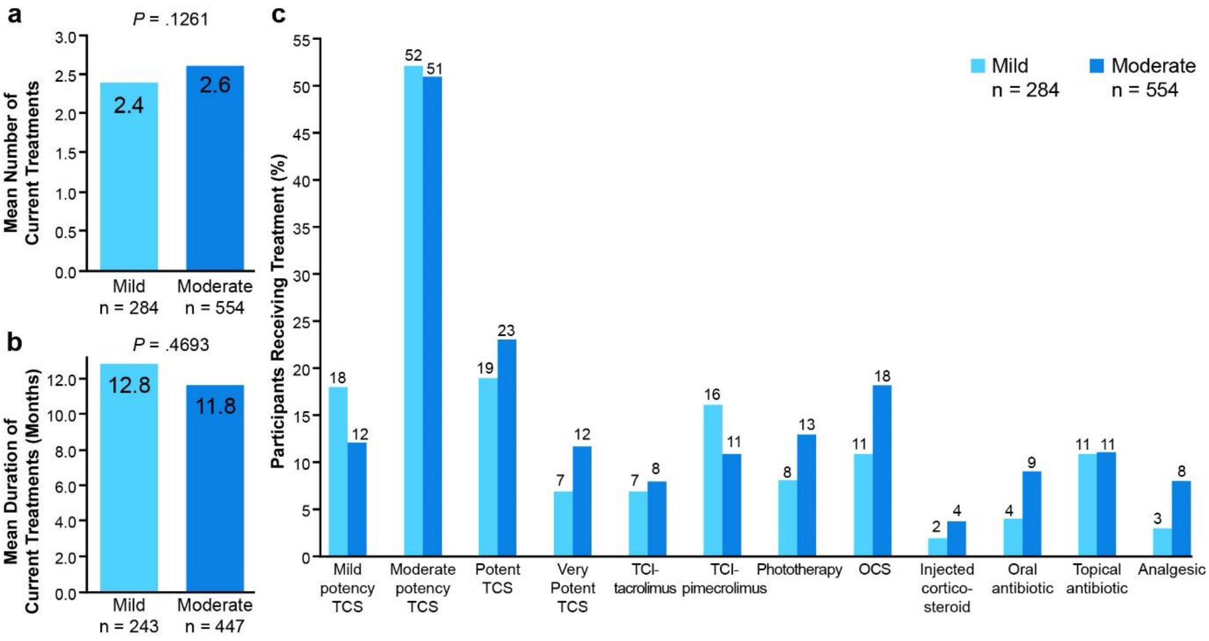

Abbreviations: OCS, oral corticosteroid; TCI, topical calcineurin inhibitor; TCS, topical corticosteroid.

eFig. S4 Current Severity Versus Severity at Initiation of Current Treatment

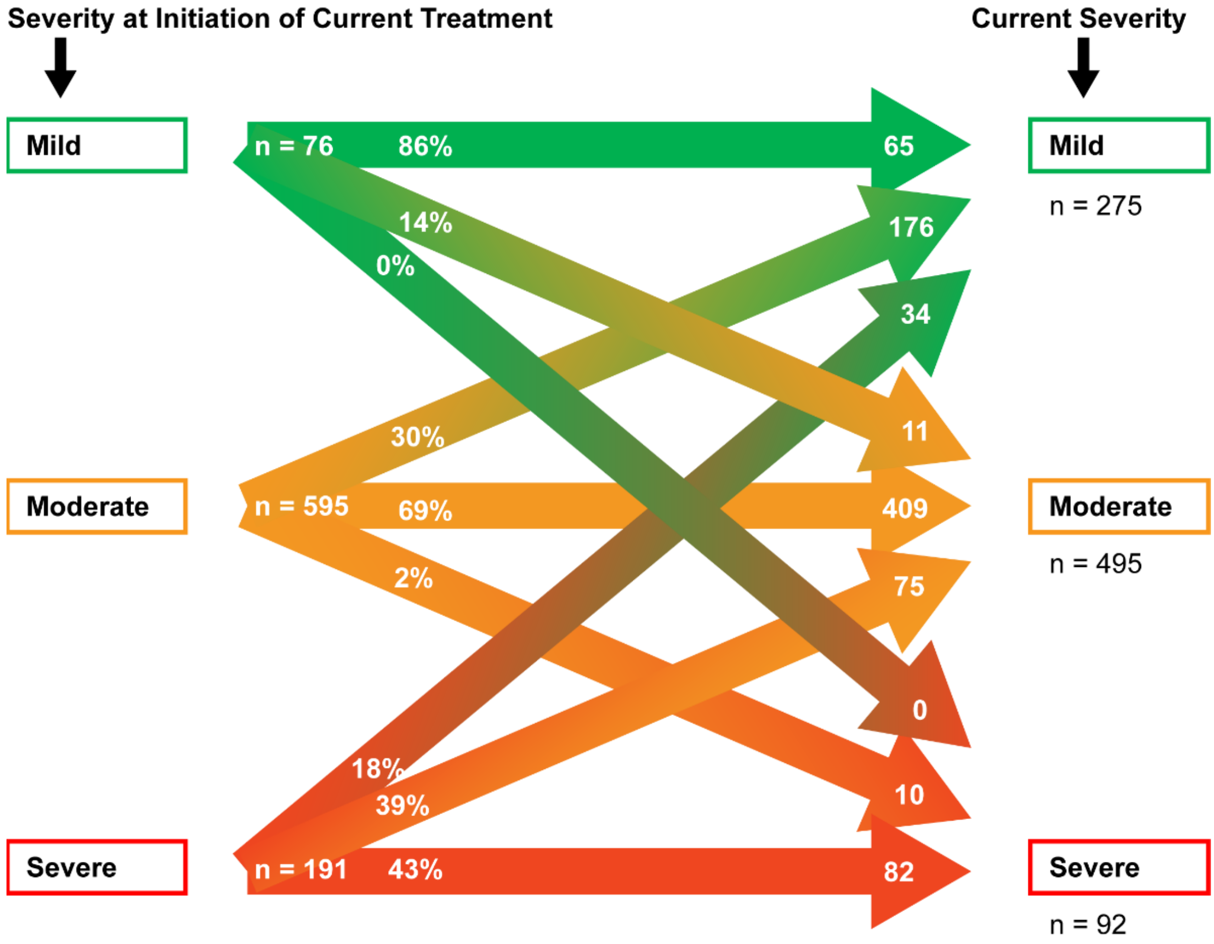

Supplement: Supplementary file 1 — Supplementary Material 1 [file 403_2025_3910_MOESM1_ESM.pdf]
